# Integrative Proteome and Acetylome Analyses of Murine Responses to *Cryptococcus neoformans* Infection

**DOI:** 10.3389/fmicb.2020.00575

**Published:** 2020-04-17

**Authors:** Hailong Li, Yanjian Li, Tianshu Sun, Wei Du, Zhijie Zhang, Dancheng Li, Chen Ding

**Affiliations:** ^1^College of Life and Health Sciences, Northeastern University, Shenyang, China; ^2^Beijing Key Laboratory for Mechanisms Research and Precision Diagnosis of Invasive Fungal Diseases, Beijing, China; ^3^Central Research Laboratory, Department of Scientific Research, Peking Union Medical College Hospital, Chinese Academy of Medical Sciences, Beijing, China; ^4^Clinical Laboratory, Shengjing Hospital of China Medical University, Shenyang, China; ^5^Software College, Northeastern University, Shenyang, China; ^6^Key Laboratory of Data Analytics and Optimization for Smart Industry, Northeastern University, Shenyang, China

**Keywords:** *Cryptococcus neoformans*, proteome, pulmonary infection, meningoencephalitis, comparative acetylome

## Abstract

*Cryptococcus neoformans* is a causative agent for pulmonary infection and meningoencephalitis. Understanding the host’s response to *C. neoformans* infection is critical for developing effective treatment. Even though some have elucidated the host response at the transcriptome level, little is known about how it modulates its defense machinery through the proteome mechanism or how protein posttranslational modification responds to the infection. In this work, we employed a murine infection model and mass spectrometry to systematically determine the proteome and acetylome statuses of lungs and brains in the early stage of infection. To extensively analyze the host response, we integrated the proteome data to the transcriptome results. Critical genes, including genes involved in phagosome, lysosome, and platelet activation are significantly altered in protein and gene expression during infection. In the acetylome analysis, we demonstrated that lung and brain tissues differentially regulate protein acetylation during infection. The three primary groups of proteins altered in acetylation status are histones, proteins involved in glucose and fatty acid metabolism, and proteins from the immune system. These analyses provide an integrative regulation network of the host responding to *C. neoformans* and shed new light on understanding the host’s regulation mechanism when responding to *C. neoformans*.

## Introduction

*Cryptococcus neoformans* is a widespread environmental human pathogenic fungus that leads to cryptococcal pneumonia and lethal cryptococcal meningitis, causing about 181,100 deaths annually (Lin and Heitman, 2006; Day et al., 2013; Perfect, 2014; Rajasingham et al., 2017). It invades the human body through the respiratory tract to the lung, and it disseminates into the brain, remaining latent until explosion. Using high-throughput transcriptome techniques, many crucial factors that play key roles in the host’s immune defense and fungal proliferation and pathogenesis have recently been identified at the host–pathogen axis (Liu et al., 2008; Chen et al., 2014). For example, using a dual RNA sequencing technique and a non-human primate infection model (Li H. et al., 2019), the host’s responsive genes were extensively mapped, and key virulence factors at the host–pathogen axis were identified. Genes of the host, including those involved in sugar metabolism and osteoblast differentiation, play vital functions against a pulmonary infection of *C. neoformans*. During early stage, *C. neoformans* lung infection, glycolysis, and citrate cycles were dampened, reducing the respiratory rate.

Traditional investigations have provided valuable information to understanding the response of a *C. neoformans* infected host, but they focused primarily on mRNA levels, neglecting the roles of proteome and protein posttranslational modification in the host (Suo et al., 2018). Protein lysine acetylation (Kac) is a conserved posttranslational modification that links acetyl–coenzyme A metabolism, including histone and non-histone acetylation (Shakespear et al., 2011). Protein posttranslational acetylation and deacetylation processes are factors in many human diseases and vital development, including neurodegenerative diseases, nerve system development, pulmonary fibrosis, neuroprogenitor survival and proliferation, neuronal maturation, maturation of astrocytes inflammation, and immunity in vertebrates (Sun et al., 2013; Choudhary et al., 2014; Li et al., 2017; Cheng et al., 2018). Numerous studies proved that the immune response was different from organ to organ (Sun et al., 2014; Tapias and Wang, 2017). In life-threatening human pathogens, lysine acetylation and deacetylation processes are critical for pathogen virulence in *Salmonella enterica*, *Mycobacterium tuberculosis*, *Candida albicans, C. neoformans*, and *Aspergillus fumigatus* (Srikantha et al., 2001; Liu et al., 2014; Sang et al., 2016; Arras et al., 2017; Brandao et al., 2018; Li Y. et al., 2019). In a recent study, comparative acetylome analysis was employed, extensively demonstrating the importance of *C. neoformans* acetylation and deacetylation processes in the regulation of fungal virulence (Li Y. et al., 2019). The deacetylases Dac2 and Dac4 participate in the balance of Kac levels in the GTP binding domain of the translation elongation factor. Moreover, both deacetylases are involved in the regulation of gene expressions of many critical virulence factors. Moreover, comparative acetylome analyses using major human fungal pathogens have demonstrated that fungal pathogens share a favorable selection of the Kac motif, indicating that acetylation site motifs co-evolve with fungal pathogenicities (Li Y. et al., 2019). Therefore, the protein Kac plays a critical role at the pathogen–host interaction, but little is known about the mechanism of the host protein Kac modulation in response to invading pathogens, and knowledge of how different tissues regulate Kac levels in response to infection is lacking.

In this study, we employed high-throughput proteome and Kac antibody enrichment acetylome mass spectrometer analyses to systematically determine the proteome and acetylome of the host’s lung and brain tissues in response to *C. neoformans* infection. To globally analyze the host’s response, we compared transcriptome and proteome data and revealed a significant overlap. We found that 127 gene products are regulated at the RNA and protein levels during pulmonary infection. Many processes involved in sugar metabolism diseases and in immune defense were significantly enriched in the proteome data. Acetylome analyses of infected host tissues were also performed, revealing that in response to *C. neoformans* infection, two major infection niches, lung and brain, showed different acetylation regulation mechanisms. This study not only serves as a fundamental host protein posttranslational modification database for infectious diseases, but it also sheds new light on deciphering the regulation of immune responses in various organs.

## Materials and Methods

### Strain and Media

*Cryptococcus neoformans* wild-type strain H99 was routinely grown in YPD agar (1% yeast extract, 2% peptone, 2% dextrose, and 2% agar). Prior to intranasal infection in mice, H99 cells were inoculated in 5 ml of YPD liquid medium and incubated at 30°C overnight. Overnight culture was then washed three times with PBS buffer and diluted in PBS. As described elsewhere, 10^5^ *C. neoformans* cells were used for infection (Suo et al., 2018; Li H. et al., 2019). For fungal burden analysis, YPD agar was used. Homogenized lung or brain tissues were diluted and plated onto YPD agar plates and were then incubated at 30°C for 2 days.

### Animal Infection

All animal experiments were reviewed and approved by the Research Ethics Committees at the College of Life and Health Sciences of Northeastern University (Approval No. 16099M). Six- to 8-week-old female mice were purchased from Changsheng Biotech (Liaoning, China) and used for infection experiments, and six mice were randomly divided into two groups of three biological replicates. Mouse care and use took place in the College of Life and Health Sciences of Northeastern University under an alternating 12-h light–dark cycle and free choice food and water. In the infection experiments, mice were anesthetized using 2,2,2-tribromoethanol, and then 10^5^ fungal cells suspended in 50 μl of PBS buffer were administered intranasally. Infected animals were inspected twice daily for morbidity. To determine fungal burden and proteome analyses, mice were humanely killed by exposure to CO_2_ for 5 min on 7 days postinfection and dissected immediately.

### Protein Extraction and Western Blotting Analysis

Upon dissection, lung or brain tissues from three uninfected and three infected animals were collected for CFU and proteome analyses. To perform proteome analysis, the brain or lung tissue was homogenized in RIPA buffer [150 mM sodium chloride, 1.0% NP-40, 0.5% sodium deoxycholate, 0.1% SDS (sodium dodecyl sulfate), 50 mM Tris, pH 8.0] using an electric homogenizer (MICCRA D-1), adding PMSF (1:40), and P8340 (1:1000) immediately before homogenization. The resultant mixture was held for 2 h at 4°C with gentle shaking, and then centrifuged at 13,000 × *g* for 20 min at 4°C. Protein samples were carefully collected from the supernatant, placed on ice, and then quantified using a BCA assay. The procedure was repeated with all three biological replicates. Lysed protein samples (30 μg) were used for SDS-PAGE and Western blot assays. Western blot assays were performed using anti-acetyllysine pan antibody (1:1000, PTM Bio, Catalog # PTM-101) and goat anti-mouse IgG (H + L) secondary antibody (1:10000, Thermo Fisher Scientific, Catalog # 31430) antibodies. The signal was captured using a ChemiDoc XRS + (Bio-Rad).

### Trypsin Digestion, Labeling, and Affinity Enrichment

Brain and lung tissue acetylome measurements were performed in biological triplicates as described elsewhere (Li Y. et al., 2019). A proteome analysis was performed first, wherein the proteins were digested with trypsin as described elsewhere (Guo et al., 2017; Li Y. et al., 2019). Briefly, tissue protein samples were collected and reduced with 5 mM DTT for 30 min at 56°C and alkylated using 11 mM iodoacetimide for 15 min at room temperature in darkness, then diluted with 0.1 M TEAB to normalize protein concentration across all samples, and then digested with trypsin (Promega, Madison, CT, United States) at 37°C overnight. After digestion, peptides were desalted using Strata X C18 SPE column (Phenomenex) and then were vacuum dried. The peptides were resuspended in 0.5 M TEAB and tandem-mass-tag (TMT-6 plex) labeling was performed to quantify the global proteome according to the manufacturer’s protocol for TMT kit. The labeled peptides were then fractionated by using an HPLC with a Thermo Betasil C18 column (5 μm particles, 10 mm × 250 mm, Thermo Fisher Scientific, United States). The fractionated peptides were dried using vacuum centrifugation and stored at −20°C until needed for further analyses. Mass spectrometry proteomics were conducted using LC-MS/MS.

To perform an acetylome analysis, the acetylated peptides were enriched by dissolving the fractionated peptides in NETN buffer (100 mM NaCl, 1 mM EDTA, 50 mM Tris–HCl, and 0.5% NP-40, pH 8.0), and then incubated with anti-Kac pan antibody-conjugated agarose beads (PTM Bio, China) at 4°C overnight with gentle shaking. The beads were washed four times with NETN buffer and twice with ddH_2_O. The bound peptides were eluted from the beads using 0.1% trifluoroacetic acid solution (Sigma, United States). The resultant peptides were combined, vacuum-dried, and then desalted using C18 ZipTips (Millipore) followed by LC-MS/MS analysis (Chen et al., 2012).

### LC-MS/MS Analysis

Liquid chromatography–tandem mass spectrometry (LC-MS/MS) was performed at PTM Biolab Hangzhou (Hangzhou, China). Peptide samples were prepared for LC-MS/MS for proteome or acetylome analyses by dissolving in 0.1% formic acid (FA) and loading directly onto a home-made reversed-phase analytical column (15 cm length, 75 μm diameter). Elution was performed using an EASY-nLC 1000 UPLC system. A 40-min gradient elution began at 6% solvent B (0.1% formic acid in 98% acetonitrile) and then increased to 23% over 26 min, to 35% over 8 min, then to 80% for 3 min, and finally held at 80% for the last 3 min. The flow rate was constant at 400 nl/min.

The resultant peptides were then analyzed using a tandem mass spectrometry (MS/MS) employing a Q Exactive^TM^ Plus hybrid quadrupole-Orbitrap mass spectrometer (Thermo Fisher Scientific, United States) coupled online to the UPLC. The electrospray voltage was set at 2.0 kV. Full MS spectra with an m/z range of 350–1800 with resolution of 70,000 in the Orbitrap were acquired. All peptides were then filtered for MS/MS using a normalized collision energy (NCE) setting of 28%. The fragments were detected in the Orbitrap at a resolution of 17,500. The electrospray voltage was again set to 2.0 kV, and the dynamic exclusion duration was set to 15 s. A data-dependent procedure that alternated between 1 MS scan and 20 MS/MS scans was performed. Automatic gain control (AGC) was set to 5E4 to prevent the ion trap overfilling (Chen et al., 2012).

### Database Search

The protein acetylation site identification and quantification were performed using the MaxQuant search engine (version 1.5.2.8) (Cox and Mann, 2008). Tandem mass spectra were searched against *Mus musculus* and *C. neoformans* var. grubii serotype A (strain H99/ATCC 208821/CBS 10515/FGSC 9487) in the UniProt database^1^, which was concatenated with a reverse decoy database (UniProt, 2019). Trypsin/P was specified as a cleavage enzyme, and up to four missing cleavages were allowed. During the primary search, a mass accuracy of ±20 ppm of the precursor ion was allowed, but results were filtered to those with mass accuracies of ±5 ppm of the precursor ions. The presence of an acetylated lysine and the mass tolerance for fragment ions was set to 0.02 Da. Carbamidomethyl on Cys was specified as a fixed modification, and acetylation modification and oxidation of Met were specified as variable modifications. Quantifications of individual proteins or acetylation sites were performed by comparing the intensities between infected and uninfected samples. The FDR was adjusted to less than 1%, and the minimum score for modified peptides was set to more than 40 (Cox and Mann, 2008; Park et al., 2013).

### Bioinformatics Analysis

Gene ontology (GO) and Kyoto Encyclopedia of Genes and Genomes (KEGG) analyses were performed using R 3.5.1, cluster profiler 3.10.1, org.Mm.eg.db (3.7.0) packages (Yu et al., 2012). The EnrichGO, enrichKEGG, and compareCluster functions were used for analyses. The protein interaction network was visualized by Cytoscape (version 3.7.1) based on the STRING database system^2^ version 11.0. The interactions that had confidence scores of at least 0.4 (medium confidence) were fetched. ClueGO was performed using ClueGO 2.5.4 with a medium network specificity (Shannon et al., 2003; Bindea et al., 2009; Szklarczyk et al., 2019).

### Data Availability

The raw acetylome and proteome mass spectrometric data have been deposited to the PRIDE Archive (Perez-Riverol et al., 2019)^3^ with identifier PXD016614.

### Statistics and Reproducibility

All experiments were performed using three biological replicates to ensure reproducibility. Sample reproducibility was analyzed using principal component analysis (PCA), performed using R, and graphs were plotted using ggplot2 (Ginestet, 2011) version 3.2.1). A correlation analysis was performed between the RNA-seq data and the proteomic data using Pearson and proteomic tests. The correlation coefficient (*r*) and a two-tailed *p* value were calculated using GraphPad Prism 7.0. The significance of process enrichment (KEGG and GO) was calculated using Clusterprofiler 3.10.1 and ClueGO 2.5.4, respectively (Bindea et al., 2009; Yu et al., 2012). After enrichment, when *p* values were less than 0.05, statistical significance was recognized. Significant alterations in protein peptides and acetylated peptides were analyzed using MaxQuant 1.5.2.8 (Cox and Mann, 2008; Tyanova et al., 2016), and when the *p* value between differentially expressed peptides was less than 0.05, statistical significance was recognized.

## Results

### Proteome and Acetylome Analyses of Lung and Brain Tissues Infected With *C. neoformans*

**FIGURE 1:**
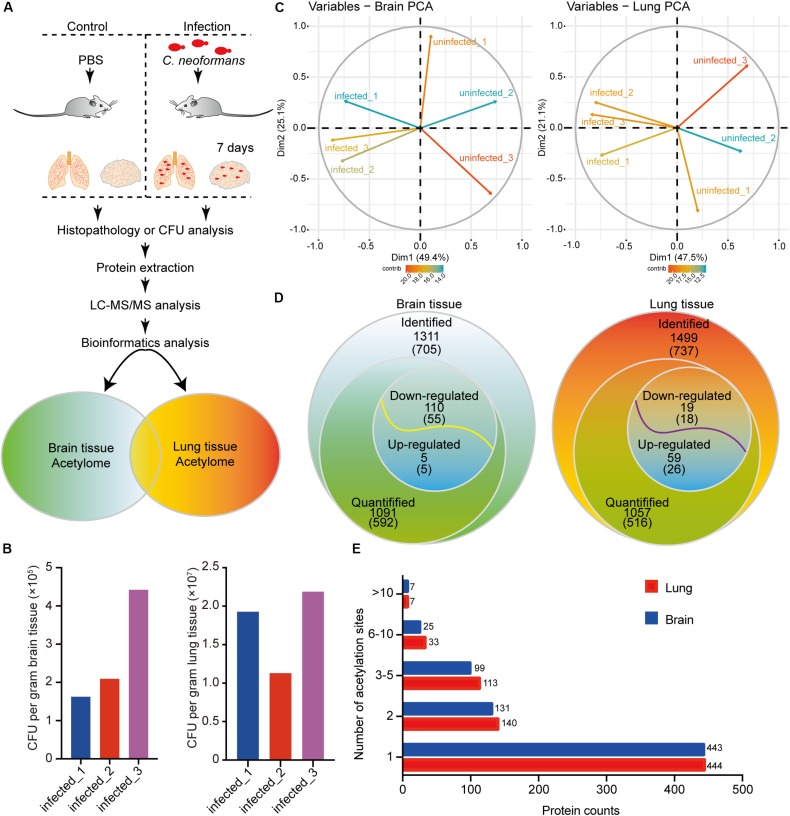
Proteome-wide identification of lysine acetylation proteins and sites in mouse brains and lungs in response to *Cryptococcus neoformans* infection. **(A)** Experimental flow chart. Six C57BL/6 female mice were divided into two groups: control group (*n* = 3) and mouse infection group (*n* = 3). Clinically isolated H99 (wild-type *C. neoformans*) were grown in YPD liquid medium overnight, and then 10^5^ fungal cells were administered intranasally to the infection group. The control group was injected with PBS buffer. At 7 days postinfection, mice were humanely killed, and brain and lung tissues were isolated for colony-forming unit (CFU) analyses, histopathological analyses, and LC-MS/MS. **(B)** CFU analyses of *C. neoformans-*infected brain and lung tissues at 7 days postinfection. At 7 days postinfection, infected animals were humanely killed, and brain and lung tissues were isolated for CFU analyses, respectively. Homogenized brain and lung tissues were diluted in PBS buffer and plated onto YPD agar media. Plated fungal cells were incubated at 30°C for 2 days, and then CFUs were calculated and normalized to brain and lung weight. **(C)** Principal component analysis (PCA) of acetylome data from lung and brain tissues. Raw data from proteome and acetylome of lung and brain tissues (three biological replicates include three uninfected control animals and three infected samples) were obtained using LC-MS/MS and were subsequentially analyzed using PCA analyses [R package ggplot2 (Ginestet, 2011) version 3.2.1]. **(D)** Survey of identified proteins and acetylation sites. The numbers of identified proteins are shown, and the corresponding identified acetylation sites are indicated in brackets. For acetylome and proteome quantification, proteins from three biological replicates with a fold change either greater (or less) than 1.3 and with *p* < 0.05 were considered as upregulated (or downregulated). **(E)** Distribution of acetylated proteins from lung and brain tissues. Identified acetylation sites were counted and plotted.

Protein acetylation is crucial for cellular biological processes. To elucidate the mechanisms of protein acetylation and to globally determine the host acetylome upon *C. neoformans* infection, we used a murine infection model, employing C57BL/6 mice, and the proteomes and acetylomes of infected lung and brain tissues were detected using LC-MS/MS (Figure 1A and Supplementary Figure S1A). Colony-forming units (CFUs) were used to validate the infiltration of *C. neoformans* cells into the brain and lung tissue, and the result indicated successful generation of fungal meningitis at 7 days postinfection (Figure 1B). Triplicate protein samples of lung and brain tissues were isolated from the control and infected groups and were then confirmed using an SDS-PAGE gel followed by immunoblotting using anti-Pan acetyllysine antibody (Supplementary Figures S1C–E).

To analyze the acetylomes of brain and lung tissues, the Kac pan antibody was employed to enrich acetylated peptides, and then MS was used to detect the Kac protein. The MS data validations are shown in Supplementary Figure S1, indicating the mass accuracy of the MS data. Additionally, PCAs were performed and showed that infected and uninfected tissues could be differentially grouped into two clusters (Figure 1C). Proteome analyses were also performed using brain and lung tissue samples (Supplementary Tables S1, S2), and these served as background controls for quantifying acetylation alterations. After comparative acetylome analysis, in the host, 1311 lysine acetylation sites from 705 proteins and 1499 sites from 737 proteins could be identified in the brain and lung, respectively, and from these, 1091 sites from 592 proteins in the brain and 1057 sites from 516 proteins in the lung could be quantified (Figure 1D and Supplementary Table S3). The number of acetylation sites found in brain tissue was comparable to that found in lung tissue (Figure 1E). Compared to uninfected tissues, 110 sites from 55 proteins were downregulated, and 5 sites from 5 proteins were upregulated in response to *C. neoformans* infection in the brain. On the other hand, 19 sites from 18 proteins were downregulated, and 59 sites from 26 proteins were upregulated in the lung (Figure 1D). Of the detected acetylated proteins, 411 were shared between the two types of tissues, and 708 Kac sites were detected in both datasets. The detected protein Kac in lung tissue was correlated to that in brain tissue in a statistically significant way: *R* = 0.699 and *p* < 0.001 (Supplementary Figure S1B). The five proteins with the most Kac sites were SPATAN (28 and 18 Kac sites in lung and brain tissues, respectively), Ncl (17 and 13 sites), EP300 (13 and 14 sites), DLD (11 and 8 sites), and MDH2 (11 and 7 sites).

### Integrative Analyses of the Host Response to *C. neoformans* Infection Using Transcriptome and Proteome Data

**FIGURE 2:**
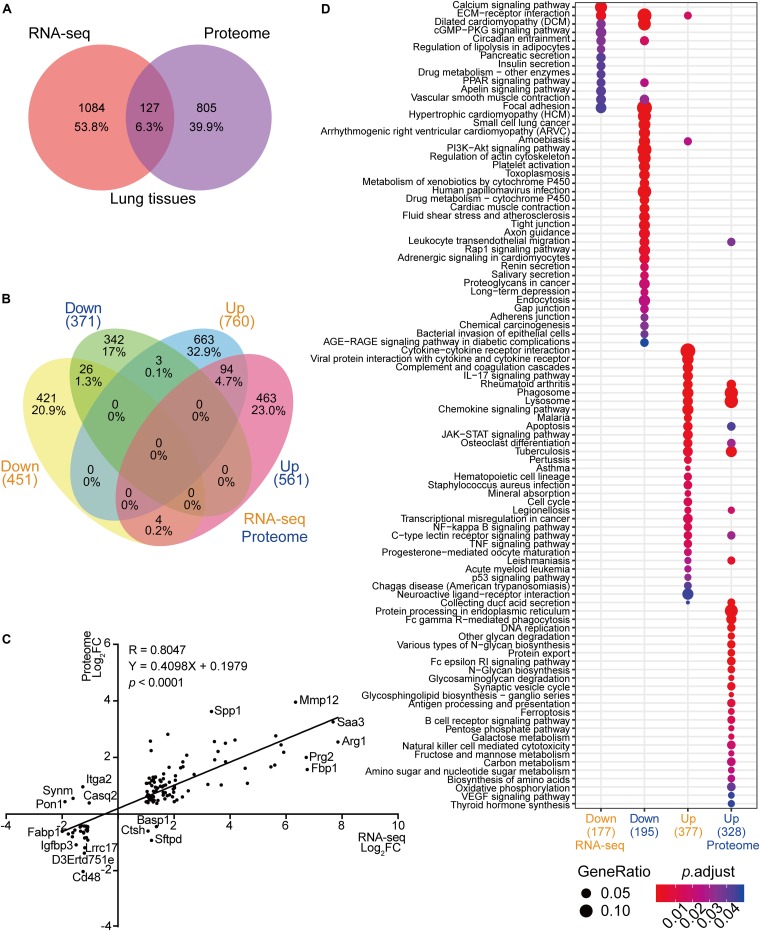
Correlation analyses between RNA-seq and proteome data from infected mice lungs. **(A)** Venn diagram of RNA-seq data and proteome data from infected lungs. RNA-seq data of infected mice lungs were obtained from https://www.ncbi.nlm.nih.gov/geo (GSE122785) (Li H. et al., 2019). Differentially expressed mRNA from the RNA-seq data and proteins from proteome data were compared. **(B)** Comparative analyses of RNA-seq and proteome data. Comparisons between RNA-seq and proteome data were performed using upregulated mRNA and protein products and downregulated mRNA and protein products. **(C)** Correlation analysis of RNA-seq and proteome data. The 127 mRNA and protein products that overlapped (Figure 2A) were subjected to a correlation analysis based on fold change and regulation pattern. The Pearson correlation coefficient (*R*) and *p* value were calculated. **(D)** Comparative Kyoto Encyclopedia of Genes and Genomes (KEGG) analyses of RNA-seq and proteome data. The KEGG analyses (https://www.genome.jp/kegg, Clusterprofiler 3.6.0) were performed using upregulated mRNAs, downregulated mRNAs, upregulated proteins, and downregulated proteins from RNA-seq and proteome data. The correlation between two datasets is demonstrated. Gene ratios are indicated by circle sizes, and when *p*_adjust_ < 0.05, a significant change in the KEGG pathway was recognized. The number of genes or proteins involved in the KEGG analyses are indicated in brackets.

In the past, transcriptome analyses using infected lung tissues have demonstrated important biological processes that were differentially expressed in response to *C. neoformans* infection (Li H. et al., 2019). To globally analyze the host molecular modulation mechanism during pulmonary infection, we systematically compared the lung transcriptome and proteome data, including comparisons between differentially expressed transcriptional mRNA species to protein products and subsequentially determining commonly differentially altered biological processes. The number of differentially expressed products detected in the transcriptome and proteome data were compared, and 127 genes were found to show significant alterations in mRNA expression and protein products across both datasets (Figures 2A,B). Of these, 94 gene products were upregulated and 26 were downregulated in both datasets. Of the remainder, three were induced in transcription but repressed in protein expression and four showed the opposite. Among the 1084 genes differentially expressed in transcription but not in protein expression, 663 were upregulated and 421 were downregulated. Finally, 805 genes showed alteration in protein expression only (Figure 2B). A Pearson correlation analysis was performed using the 127 genes shared between the two datasets, revealing a significant correlation (*R* = 0.8047; *p* < 0.001) (Figure 2C). The mRNAs and proteins of *MMP12*, *SAA3*, and *ARG1* were robustly induced in both the transcriptome and proteome datasets. The seven gene products that were reciprocally regulated were *ITGA2*, *SYNM*, *PON1*, *CASQ2*, *BASP1*, *CTSH*, and *SFTPD.*

Even though limited gene products were shared between the transcriptome and proteome analyses, the GO and the KEGG analyses revealed substantial overlaps in biological processes critical for battling invading pathogens. The processes showing significant enrichment in both datasets were amebiasis, tuberculosis, phagosome, lysosome, HIF1-signaling pathway, osteoclast differentiation, and PPAR-signaling pathways (Figure 2D and Supplementary Figure S2).

**FIGURE 3:**
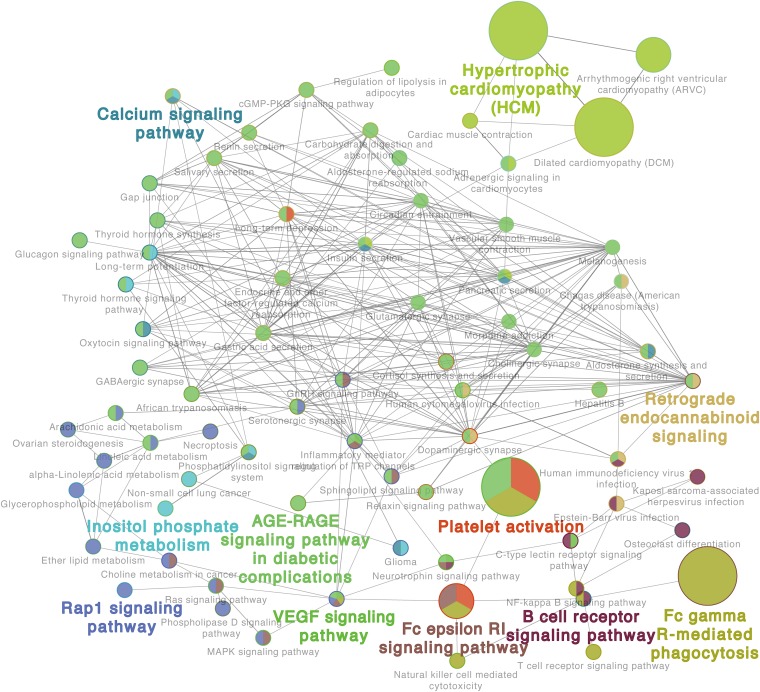
Proteome gene ontology (GO) analyses of infected lung tissues. The GO reactome analysis was performed using differentially expressed proteins in lung tissues. A representative GO result is shown. Additional results are shown in Supplementary Figure S3.

Additionally, the GO analysis using lung proteome data revealed a regulatory network in the response to *C. neoformans* infection. It included the calcium signaling pathway, platelet activation, B-cell receptor signaling pathway, Fc gamma R-mediated phagocytosis, and VEGF signaling pathways, all significantly enriched in lung tissue (Figure 3). Other GOs were enriched as well, but interaction networks were not involved. These GO terms were divided into three major groups: disease pathways (including pathways involved in asthma, *Salmonella* infection, bacterial invasion of epithelial cells, and the insulin signaling pathway), amino acid and sugar metabolism (including processes involved in glycolysis, fatty acid degradation, and amino acid metabolisms), and immune responses (including pathways involved in leukocyte transendothelial migration, the toll-like receptor signaling pathway, and the chemokine signaling pathway) (Supplementary Figure S3). These data demonstrate the significant correlation between transcriptome and proteome levels in lung tissue infected with *C. neoformans*, and it provides a global understanding of the host response to *C. neoformans* pulmonary disease.

Thus far, the sensitivity property of the RNA-seq technique has been used to comprehensively measure the simultaneous or dual transcriptome responses in invading pathogens and their hosts, providing a rapid detection of transcription alteration at the host–pathogen axis (Nuss et al., 2017; Li H. et al., 2019). The proteome peptides were searched against the *C. neoformans* proteome database. In total, 66 peptides corresponding to 45 proteins were detected in brain samples and 107 peptides corresponding to 66 proteins were detected in lung samples (Supplementary Tables S1, S2). However, these peptides or proteins were also detected in protein samples from the uninfected animals, those that lacked *C. neoformans* cells. This implies that these 94 proteins are homologous proteins from mice. A similar phenomenon was observed in the acetylome analyses (Supplementary Table S3). Taken together, the proteome and acetylome analyses are far less sensitive compared to the RNA-seq technique, to allow the performance of dual proteome or acetylome analyses.

### *C. neoformans* Infection Modulates Platelet Activation

**FIGURE 4:**
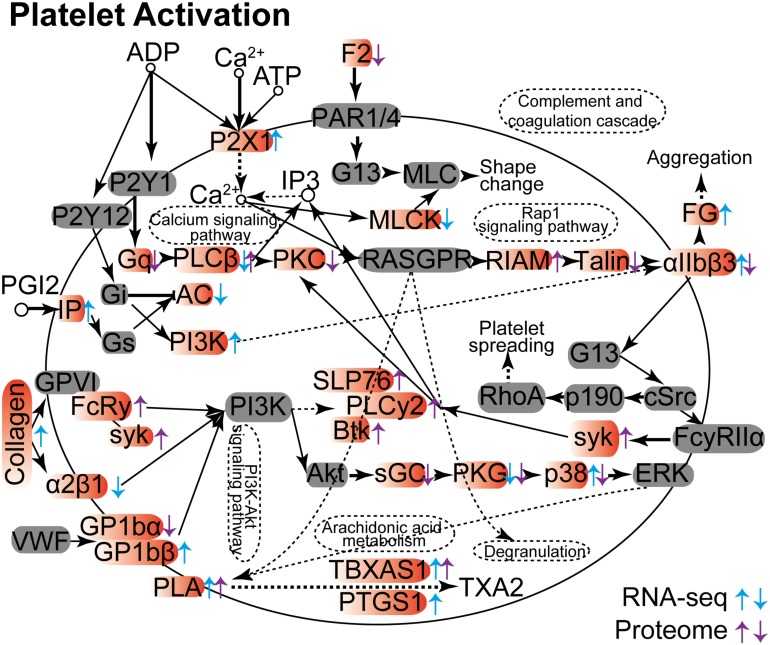
Platelet activation Kyoto Encyclopedia of Genes and Genomes analyses between RNA-seq and proteome data. The differentially expressed mRNA and protein products are indicated by arrows, with upward arrows indicating upregulation and downward arrows indicating repression. Genes from RNA-seq data are shown in blue, and proteins from proteome data are shown in purple.

Others have demonstrated that platelet activation plays a key role against invading viruses, bacteria, and fungi in the host. Included are cytomegalovirus infection, *Helicobacter pylori* infection, and *Aspergillus* infection (Elizalde et al., 1997; Perkhofer et al., 2008; Soroceanu et al., 2008; Souza et al., 2009). We show here that the platelet activation process in lung tissue is dramatically influenced in both RNA and protein levels upon *C. neoformans* infection (Figure 4). However, gene products could be differentially regulated in RNA or protein levels. For example, PKG (prkg1 expression was modulated in its RNA level, and prkg1 and prkg2 were expressed differently in their protein levels), TBXAS1 (tbxas1 expression was altered in both RNA and protein levels), and PLA (pla2g4c was changed in its RNA level, and pla2g4a and pla2g4c were changed in their protein levels) were regulated uniformly between their RNA and protein levels. However, some gene products demonstrated reciprocal regulation patterns. Examples are αIIbβ3 (itga2b was upregulated in its RNA level but downregulated in its protein level), PLCβ (plcb1 was downregulated in its RNA level, and plcb2 was upregulated in its protein level), and p38 (papk13 was upregulated in its RNA level, and mapk12 was downregulated in its protein level). On the other hand, some genes were regulated either in their RNA levels or in their protein levels, mostly as a result of posttranslational modification. Despite these differences, pathway-associated infections and immunity were enhanced by platelet activation in both RNA and protein levels, including calcium signaling pathways, complementing the coagulation cascade, aggregation, rap1 signaling pathways, PIK3-Akt signaling pathways, arachidonic acid metabolism, and degranulation pathways.

### Acetylome Analyses of Brain and Lung Tissues Infected With *C. neoformans*

**FIGURE 5:**
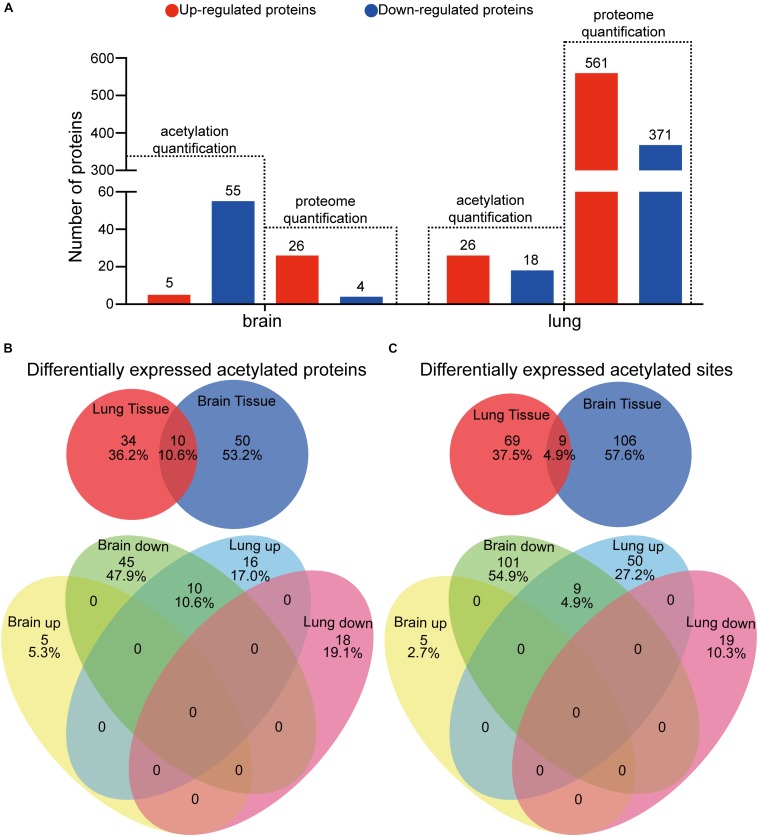
Analyses of differentially expressed protein lysine acetylation (Kac) proteins in response to *Cryptococcus neoformans* infections. **(A)** Comparative analyses between proteome and acetylome of infected lung and brain tissues. Differentially expressed proteins or protein Kac sites with fold changes greater than 1.3 and with *p* < 0.05 are considered significant. **(B)** Comparative analysis of differentially expressed Kac proteins between infected lung and brain tissues. **(C)** Comparative analysis of differentially expressed Kac sites between infected lung and brain tissues.

To decipher the influence of protein lysine acetylation in response to *C. neoformans* in brain and lung tissues, we quantified the acetylated proteins and acetylation sites. Proteins or sites with *p* < 0.05 and fold changes either greater than 1.3 or less than 0.769 compared to the control group (uninfected mice) were considered significantly changed. We found differential expressions of 60 and 44 acetylated proteins in brain and lung tissues, respectively, corresponding to 115 and 78 Kac sites (Figures 5A, 1D). Fifty-five Kac proteins were downregulated and five were upregulated in infected brain tissue, while 26 Kac proteins were upregulated and 18 were downregulated in infected lung tissue. Of the set of differentially expressed acetylated proteins from lung and brain tissues, only 10 were co-detected: histone H3.3, histone H4, Sptan1, Prkar2a, Prdx6, Sptbn1, Dlst, Septin11, Tppp3, and Hadh. However, all Kac sites from these proteins were oppositely regulated between the two types of tissues, specifically, downregulated in infected brain tissue and upregulated in infected lung tissue (Figures 5B,C), indicating the unique regulation patterns of these acetylation sites between the lung and brain. Furthermore, 115 Kac sites were significantly modulated in brain tissue, 110 downregulated and 5 upregulated, and 78 were differentially expressed in infected lung tissue, 19 repressed and 59 induced. The Venn diagram shows that both types of infected tissues share nine Kac sites: histone H3.3 (K28ac), histone H4 (K6ac), Sptan1 (K1939ac and K2349ac), Prdx6 (K63ac, K106ac, and K215ac), Sptbn1 (K1824ac), and Tppp3 (K136ac) (Supplementary Figure S4). These shared Kac sites were repressed in brain tissue but induced in lung tissue (Figure 5B).

**FIGURE 6:**
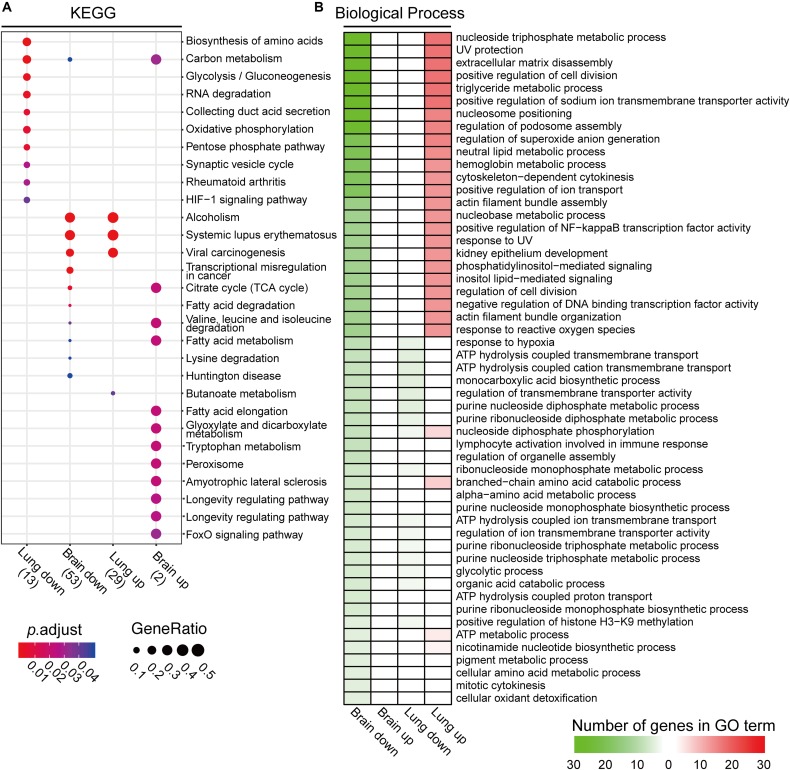
Kyoto Encyclopedia of Genes and Genomes (KEGG) and gene ontology (GO) analyses of proteins with differentially expressed protein lysine acetylation (Kac) proteins from infected brain and lung tissues. **(A)** Comparative KEGG analyses of differentially expressed Kac proteins from infected brain and lung tissues. When *p*_adjust_ < 0.05, significant enrichment in the KEGG process was recognized. **(B)** Gene ontology analysis of Kac proteins from *Cryptococcus neoformans-*infected lung and brain tissues. The GO analysis was performed using Clusterprofiler 3.6.0. Significantly enriched GO terms are shown. Upregulated GO processes are indicated in red, and downregulated GO processes are shown in green.

The KEGG analyses, using differentially expressed brain and lung acetylated proteins, showed that the pathways of alcoholism, systemic lupus erythematosus, and viral carcinogenesis are reciprocally regulated between brain (downregulation) and lung tissues (upregulated) (Figure 6A). Carbon metabolism was also enriched in both tissues, again showing opposite regulation patterns. In infected brain tissue, the FoxO signaling pathway, longevity regulating pathway, longevity regulating pathway-multiple species, amyotrophic lateral sclerosis, peroxisome, tryptophan metabolism, glyoxylate and dicarboxylate metabolism, and fatty acid elongation were significantly enriched (upregulation). On the other hand, pathways including Huntington disease, lysine degradation, valine, leucine and isoleucine degradation, and transcriptional misregulation in cancer were downregulated. Sugar and lipid metabolism including fatty acid metabolism, fatty acid degradation, and the citrate cycle were changed in mouse brain tissue. In lung tissue, downregulated pathways were the HIF-1 signaling pathway, rheumatoid arthritis, synaptic vesicle cycle, pentose phosphate pathway, oxidative phosphorylation, collecting duct acid secretion, RNA degradation, glycolysis/gluconeogenesis, and the biosynthesis of amino acids, while the upregulated pathway was butanoate metabolism (Figure 6A).

The GO analyses showed drastic differences in the biological processes of differentially expressed acetylated proteins between infected brain and lung tissues (Figure 6B and Supplementary Figure S5). Major processes included the nucleoside triphosphate metabolic process, UV protection, extracellular matrix disassembly, positive regulation of cell division, the triglyceride metabolic process, positive regulation of sodium ion transmembrane transporter activity, nucleosome positioning, regulation of podosome assembly, regulation of superoxide anion generation, and the neutral lipid metabolic and hemoglobin metabolic processes. Also included were cytoskeleton-dependent cytokinesis, positive regulation of ion transport, actin filament bundle assembly, the nucleobase metabolic process, positive regulation of NF-kappa B transcription factor activity, response to UV, kidney epithelium development, phosphatidylinositol-mediated signaling, inositol lipid-mediated signaling, regulation of cell division, negative regulation of DNA binding transcription factor activity, actin filament bundle organization, and response to reactive oxygen species. The protein-DNA complex, nucleosome, DNA packaging complex, nuclear chromatin, and nuclear nucleosome are the most-changed cellular component pathways between the two organs (Supplementary Figure S5). Taken together, these data demonstrate profound differences in acetylome between two niches for infection, suggesting that acetylation in brain and that in the lung play reciprocal functions during *C. neoformans* infection.

### Reactome Network Construction of Differentially Expressed Kac Proteins

**FIGURE 7:**
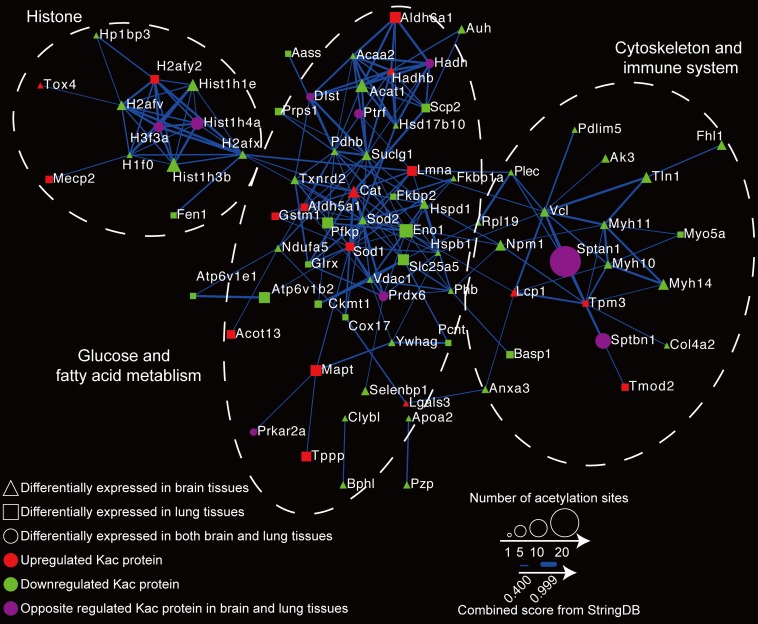
Reactome network of proteins with differentially expressed protein lysine acetylation (Kac) sites in response to *Cryptococcus neoformans* infection. The relationship of differentially expressed Kac protein was calculated using the STRING database (see text footnote 2), and the reactome network was constructed. Differentially expressed Kac proteins from brain tissues are indicated by triangles; those from lung tissues are indicated by rectangles, and those from both are indicated by circles. Upregulated protein Kac sites are shown in red, downregulated Kac sites are shown in green, and Kac sites that are oppositely regulated between two are shown in purple. The STRING database scores are indicated by line weight.

To globally map the protein–protein interactions between differentially expressed Kac proteins in the host, we executed a PPI network analysis using the STRING database (see text footnote 2) (Szklarczyk et al., 2019). In the reactome network, 94 differentially expressed acetylated proteins were employed for PPI. Of those, 81 (with 207 interactions) were searched, and the network was generated (Figure 7 and Supplementary Table S4). The acetylated proteins associated with brain and lung tissues can generally be divided into three groups: the histone group, the glucose and fat metabolism group, and the cytoskeleton and immune system group. These proteome reactome networks reveal that *C. neoformans* infection modulates the acetylation status of histones and changes the chromatin transcription rate, which in turns regulates sugar metabolism and immune system expressions in the host.

## Discussion

We previously determined the response of *C. neoformans* infection at the transcriptome level in a murine model (Li H. et al., 2019). However, proteome and protein posttranslational modifications are highly dynamic events, and most importantly, the transcriptome responses do not necessarily correlate and reflect that status of the proteome and PPTMs. To map the host’s global response to *C. neoformans*, we performed the proteome analysis on tissues from two important organs, the lung and the brain (Lin and Heitman, 2006). Comparative results between the transcriptome and proteome revealed a substantial number of common genes involved in ECM–receptor interaction, phagosome, lysosome, osteoblast differentiation, and tuberculosis (Li H. et al., 2019). The extent of these genes suggests that some processes are regulated at both the RNA and protein levels, yet a clearly different pattern of regulation exists between RNA and protein products. A commonly regulated product at the RNA and protein levels is MMP12. However, we have demonstrated that induced MMP12 plays no function in protecting the host against *C. neoformans* infection (Li H. et al., 2019). An osteoblast key play, OCSTAMP, was induced in the transcriptome data, and CRISPR-Cas9-mediated knockout mice showed enhanced resistance to *C. neoformans*. However, the protein expression of OCSTAMP was not significantly altered in the lung proteome. One possible scenario is that the protein status is controlled at many levels, including the protein translation rate and protein proteasome and autophagy degradations. Interestingly, the OCSTAMP protein is predicted to consist of four possible ubiquitination sites: K116, K192, K289, and K335^4^. Therefore, it is most likely that the protein product is quickly degraded even though the gene expression is highly induced, thus resulting in a non-significant change in protein level (Hershko and Ciechanover, 1992).

In this study, we aimed to address another critical goal: to identify and map the acetylation status in response to *C. neoformans* infection. Two organs, in response to one invading pathogen, somehow demonstrated drastic and reciprocal responses in the acetylome. Most of the Kac proteins and Kac sites were downregulated during meningitis formation, yet pulmonary infection induced the expression of Kac proteins and sites in lung proteins. The comparative analysis between brain and lung tissues for Kac status revealed no commonly regulated targets, suggesting that the regulation mechanisms of acetylation and deacetylation processes differed between the two types of tissues.

Lines of evidence have shown different tissue responses between these organs in response to *C. neoformans* infection, including differentially regulated Cu homeostasis and diverged transcriptome responses (Sun et al., 2014; Forrester et al., 2018; Li C. et al., 2019). For example, *C. neoformans* senses reciprocal Cu environments in the lung and brain, and it turns on expression of Cu detoxification and acquisition mechanisms, respectively. We recently identified over 1000 transcripts differentially expressed in lung tissue during pulmonary *C. neoformans* infection, while only 20 differentially expressed genes were detected in brain tissue (Li H. et al., 2019). In this work, we showed that proteome and acetylome responses are also differentially regulated. The regulation mechanism that results in the different responses remains unclear. These data suggest the necessity of analyzing host niches when studying a host’s response to *C. neoformans* infection.

In conclusion, this work provides comprehensive proteome and acetylome data of *C. neoformans* for infected brain and lung tissues in a murine model, and it highlights the importance of lysine acetylation during infections and elucidates differences in Kac status between brain and lung tissues. Our data shed new light on a new conceptual understanding of the immune differences between CNS and other tissues.

## Data Availability Statement

The datasets generated for this study can be found in the raw acetylome and proteome mass spectrometric data have been deposited to the PRIDE Archive (https://www.ebi.ac.uk/pride) with identifier PXD016614.

## Ethics Statement

The animal study was reviewed and approved by the Research Ethics Committees at the College of Life and Health Sciences of Northeastern University (Approval No. 16099M).

## Author Contributions

CD conceived the project. TS, YL, ZZ, and CD designed the study. YL and TS performed the mouse infection experiments. HL, WD, DL, and CD participated in the data analysis. HL and CD composed the manuscript.

## Conflict of Interest

The authors declare that the research was conducted in the absence of any commercial or financial relationships that could be construed as a potential conflict of interest.

## Footnotes

1 http://www.uniprot.org

2 http://string-db.org/

3 https://www.ebi.ac.uk/pride

4 http://csb.cse.yzu.edu.tw
