# Supplementary material for: Integrative Proteome and Acetylome Analyses of Murine Responses to Cryptococcus neoformans Infection
Source: Front Microbiol. 2020 Apr 17;11:575. doi: 10.3389/fmicb.2020.00575 (PMC7181412; doi:10.3389/fmicb.2020.00575)
Supplement: Supplementary file 1 [file Data_Sheet_1.docx]

**Supplemental Figure 1. Proteome analysis of *Cryptococcus neoformans* infected lung and brain tissues.**

1. Dot plot of peptide score and mass error of brain and lung tissues. The distribution of the mass error of brain and lungs tissues are plotted. Each plot indicates three biological replicates.
2. Correlation of protein lysine acetylation sites between brain and lung tissues. The correlation was calculated based on the proteins in Figure 1D. The calculated correlation coefficient is shown.
3. Coomassie Brilliant Blue staining of proteins isolated from brain and lung tissues.
4. Immunoblotting of brain protein samples using anti-pan acetyllysine antibody.
5. Immunoblotting of lung protein samples using anti-pan acetyllysine antibody.

**Supplemental Figure 2. Comparative analyses of RNA-seq and proteome data of lung and brain.**

1. Venn diagram of transcriptome and proteome of infected brain tissues.
2. The gene ontology (GO) Venn diagram of transcriptome and proteome of infected lung tissues.
3. The GO reactome network of transcriptome and proteome of infected lung tissues.

**Supplemental Figure 3. Gene ontology (GO) analyses of differentially expressed proteins in lung proteome.**

The GO reactome analysis of proteins with differentially expressed protein lysine acetylation sites in lung tissues.

**Supplemental Figure 4. MS/MS spectra of acetylation sites shared between infected brain and lung tissues**

1. MS/MS spectrum of peptide K(ac)SAPSTGGVK in histone H3.3 (K28ac).
2. MS/MS spectrum of peptide GK(ac)GGK(ac)GLGK(ac)GGAK(ac)R in histone H4 (K6ac).
3. MS/MS spectrum of peptide K(ac)NNHHEENISSK in Sptan1 (K1939ac).
4. MS/MS spectrum of peptide LNHQEFK(ac)SCLR in Sptan1 (K2349ac).
5. MS/MS spectrum of peptide LAPEFAK(ac)R in Prdx6 (K63ac).
6. MS/MS spectrum of peptide LPFPIIDDK(ac)GR in Prdx6 (K106ac).
7. MS/MS spectrum of peptide ELPSGK(ac)K in Prdx6 (K215ac).
8. MS/MS spectrum of peptide IQDK(ac)HKK in Sptbn1 (K1824ac).
9. MS/MS spectrum of peptide YTGSHK(ac)ER in Tppp3 (K136ac).

**Supplemental Figure 5. Comparative gene ontology (GO) analysis of differentially expressed proteins between infected lung and brain tissues.**

The GO analysis of proteins with differentially expressed protein lysine acetylation proteins in lung tissues. Cellular component and molecular function GO analyses are shown.

**Supplemental Table 1.** Proteome from brain tissues during *Cryptococcus neoformans* infections.

**Supplemental Table 2.** Proteome from lung tissues during *Cryptococcus neoformans* infections.

**Supplemental Table 3.** List of identified lysine acetylated proteins and sites from brain and lung tissues.

**Supplemental Table 4.** Protein-protein interaction network for all acetylated proteins.
