# Supplementary figures and images for: Integrative Proteome and Acetylome Analyses of Murine Responses to Cryptococcus neoformans Infection
Source: Front Microbiol. 2020 Apr 17;11:575. doi: 10.3389/fmicb.2020.00575 (PMC7181412; doi:10.3389/fmicb.2020.00575)

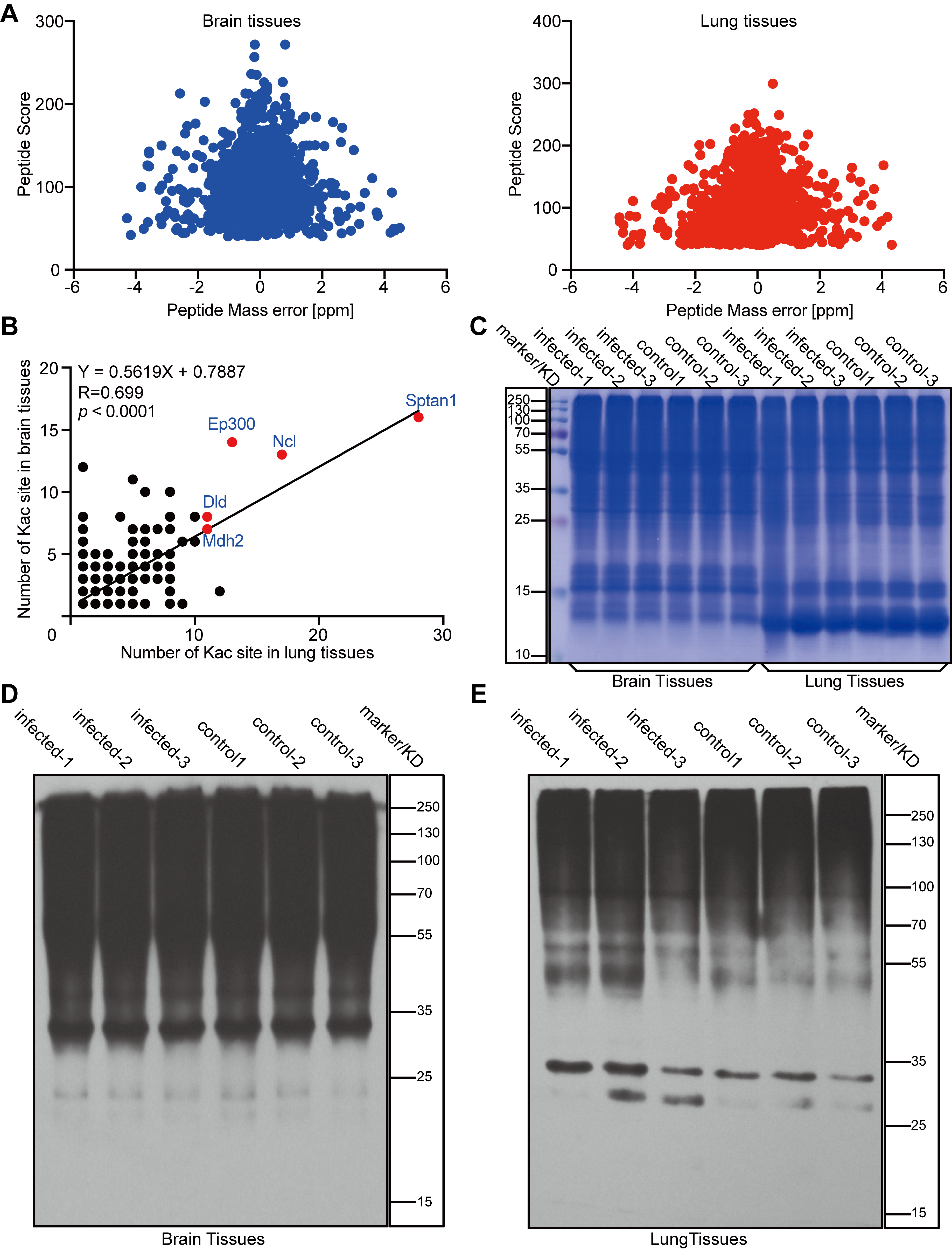

Supplement: Supplementary file 2 [file Image_1.TIF]

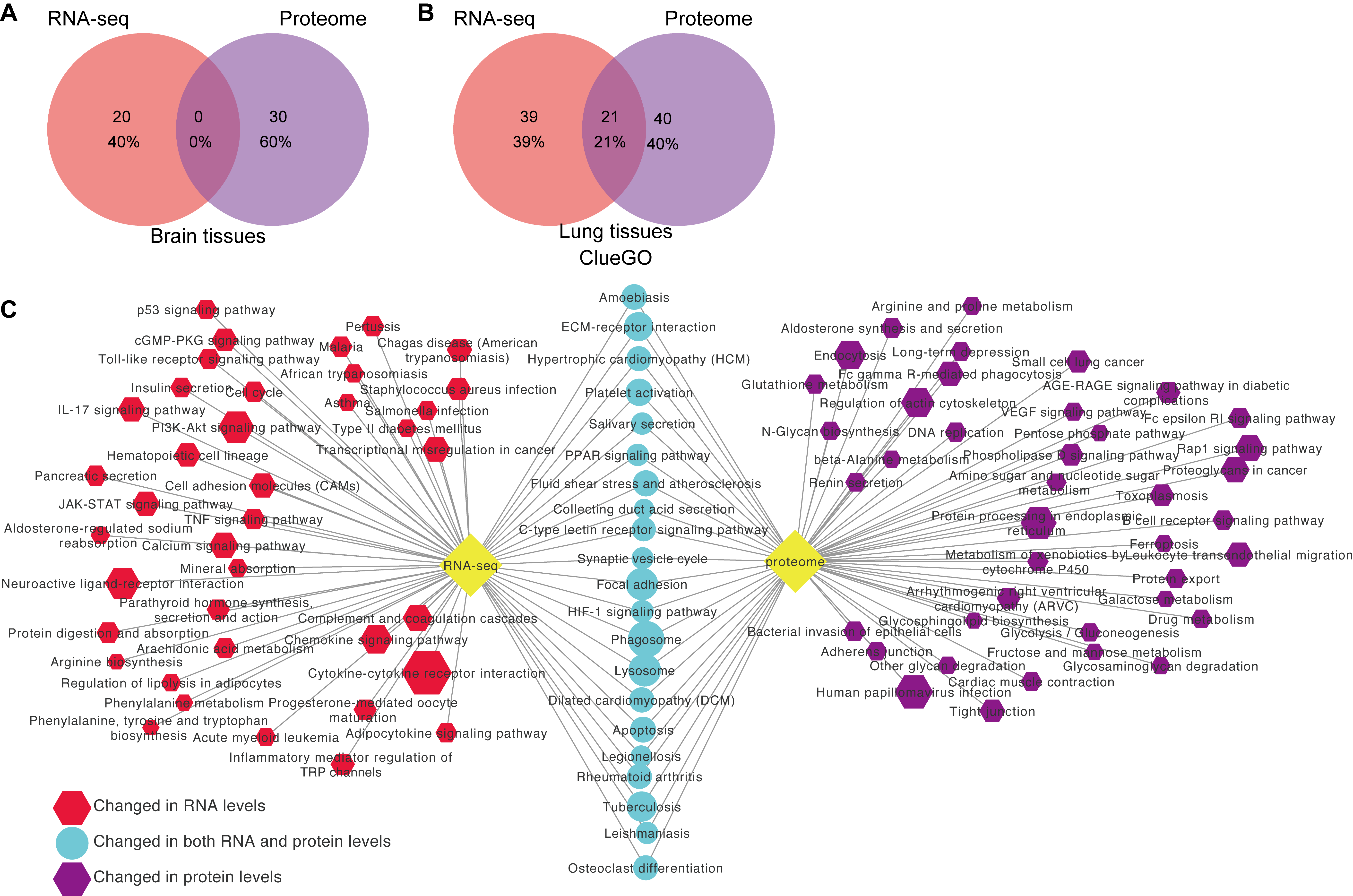

Supplement: Supplementary file 3 [file Image_2.TIF]

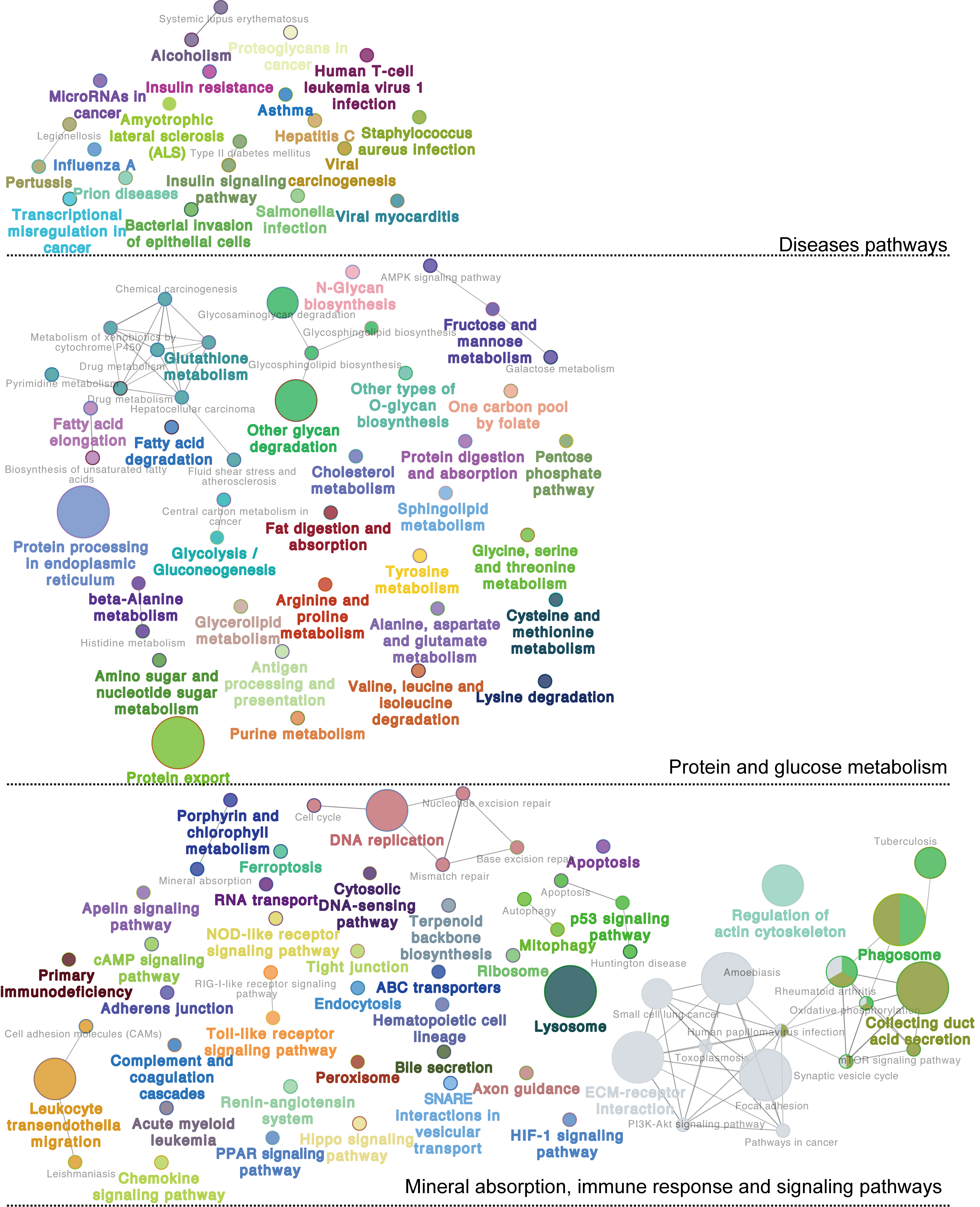

Supplement: Supplementary file 4 [file Image_3.TIF]

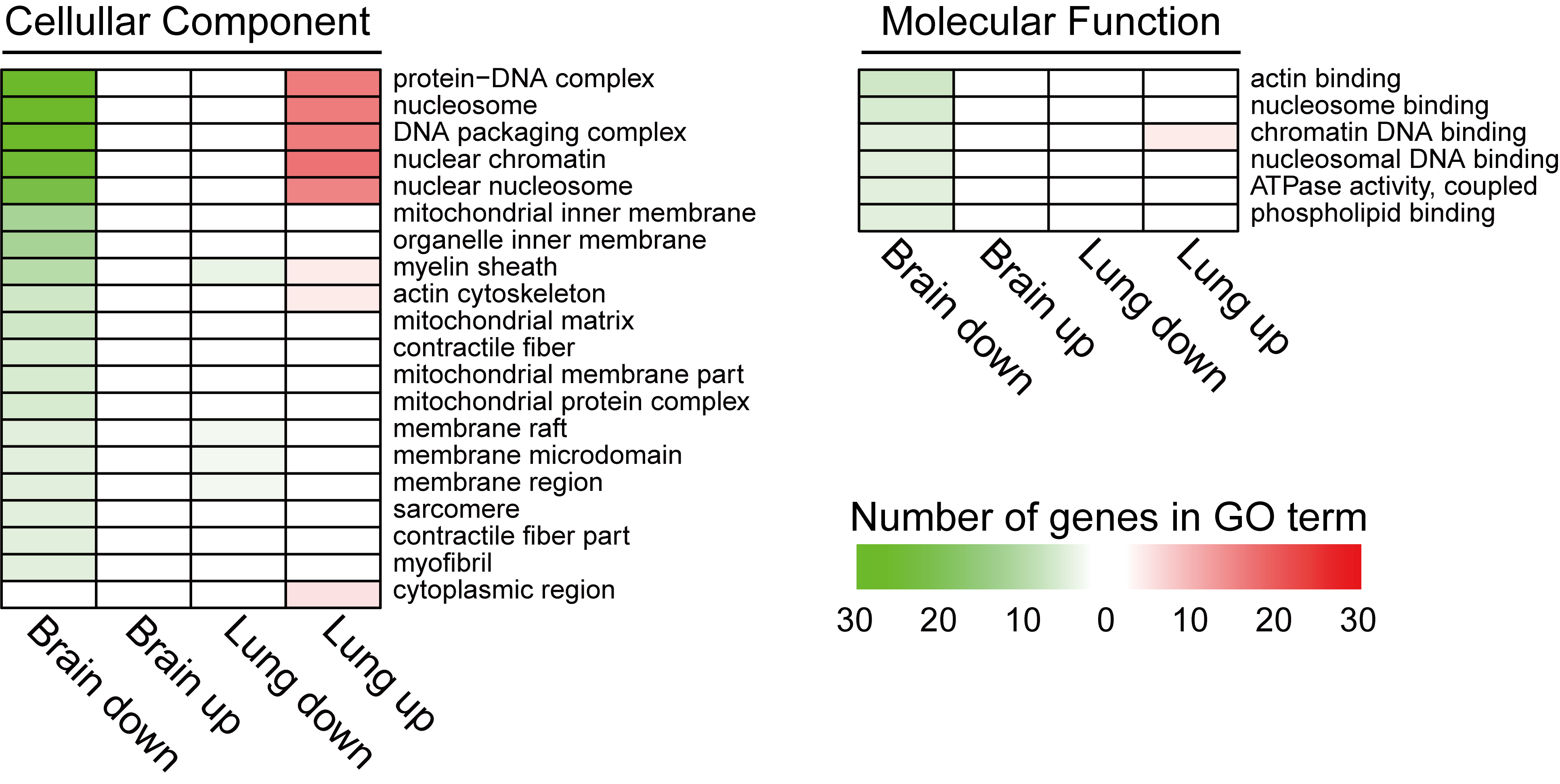

Supplement: Supplementary file 6 [file Image_5.TIF]
